# Uncovering the Roles of MicroRNAs in Major Depressive Disorder: From Candidate Diagnostic Biomarkers to Treatment Response Indicators

**DOI:** 10.3390/life11101073

**Published:** 2021-10-11

**Authors:** Claudia Homorogan, Diana Nitusca, Edward Seclaman, Virgil Enatescu, Catalin Marian

**Affiliations:** 1 Department of Biochemistry, University of Medicine and Pharmacy Victor Babes Timisoara, 300041 Timișoara, Romania; homorogan.claudia@umft.ro (C.H.); nitusca.diana@umft.ro (D.N.); eseclaman@umft.ro (E.S.); 2 Discipline of Psychiatry, Department of Neurosciences, University of Medicine and Pharmacy Victor Babes Timisoara, 300041 Timisoara, Romania; enatescu.virgil@umft.ro; 3 Eduard Pamfil Psychiatric Clinic, Timisoara County Emergency Clinical Hospital, 300041 Timisoara, Romania

**Keywords:** major depressive disorder, microRNAs, biomarkers, antidepressant treatment

## Abstract

Major depressive disorder (MDD) is a recurrent debilitating illness that represents a major health burden due to its increasing worldwide prevalence, unclear pathological mechanism, nonresponsive treatment, and lack of reliable and specific diagnostic biomarkers. Recently, microRNA species (miRs) have gained particular interest because they have the ability to post-transcriptionally regulate gene expression by modulating mRNA stability and translation in a cohesive fashion. By regulating entire genetic circuitries, miRs have been shown to have dysregulated expression levels in blood samples from MDD patients, when compared to healthy subjects. In addition, antidepressant treatment (AD) also appears to alter the expression pattern of several miRs. Therefore, we critically and systematically reviewed herein the studies assessing the potential biomarker role of several candidate miRs for MDD, as well as treatment response monitoring indicators, in order to enrich the current knowledge and facilitate possible diagnostic biomarker development for MDD, which could aid in reducing both patients’ burden and open novel avenues toward a better understanding of MDD neurobiology.

## 1. Introduction

Major depressive disorder (MDD) is a complex neuropsychiatric disorder with an increasing incidence and a 2–20% prevalence in the worldwide general population [1], being the leading cause of disability around the world [2]. A significant decrease in life quality, functional impairment, and other psychosocial aspects, as well as comorbidities are associated with MDD, among others. What is more, a high degree of disability, morbidity, and mortality by suicide (suicidal ideation) causes MDD to be considered a major public health concern in developed countries [3].

Although tremendous efforts have been made in order to better understand and characterize this debilitating illness, current knowledge regarding MDD pathophysiology and neurobiology have failed to completely elucidate its molecular particularities to a greater extent. As a consequence, about 40% of patients with MDD do not respond to antidepressant treatment (AD) and eventually become treatment-resistant as the disease burden increases [4]. In addition, although being diagnosed at relatively early ages in a somewhat efficient fashion, the lack of uniform and accurate diagnostic tools (biomarkers) may lead to difficulties in assessing the differences between MDD and other etiologically related diseases, such as bipolar disorder (BD) [5]. Performing the Diagnosis and Statistical Manual of Mental Disorders (DSM-5) and the 11th Revision of the International Classification of Diseases (ICD 11) as the gold standard diagnostic criterion applied to patients was shown to induce interviewer bias, especially if performed by only one health specialist, which might lead to misdiagnosis in many cases [6]. Moderate reliability has been attributed to the Structured Clinical Interview for DSM-IV Axis I Disorders as well (SCID-I) [7,8,9].

To date, it is known that MDD patients suffer multiple alterations in different regions of the brain, compared to healthy subjects. Studies have shown that qualitatively, synaptic circuits and neural, functional, and structural plasticity are steadily impaired, while connectivity between different brain regions is disrupted. The latter affects communication between subcortical areas involved in modulating negative emotions, the frontal lobe with other brain regions, ultimately affecting cognition, memory, and learning [10,11,12]. Evidence reveals that MDD subjects present a smaller hippocampal volume, a modified morphology (number and shape) of dendrites, and the atrophy of neurons [13,14,15,16,17,18].

Thus, it is becoming increasingly clear that MDD arises primarily due to systematic alterations in the intimate biochemical and signaling pathways involved in mood, cognition, and disposition. Impaired and/or compromised cellular networks involved in MDD remain to be understood and are extensively being investigated in modern research, which has the ability to fine-tune the current approaches and the understanding of MDD neurobiology. However, mounting evidence suggests that no single mechanism can entirely account for and encompass the etiopathogenesis of MDD [11].

Recently, studies of microRNAs (miRs) have been given great attention for their potential role in the etiology and pathophysiology of mental disorders, such as MDD. miRs are small (18–25 nucleotides in length) endogenous noncoding RNA molecules, which post-transcriptionally regulate gene expression by complementarily binding to the 3’UTR regions of target messenger RNAs (mRNAs), ultimately leading either to mRNA degradation or translation inhibition [19,20]. Literature reports have shown that miRs regulate gene expression in a cohesive fashion, being virtually involved in all biological functions [21]. They have been observed to play key roles in physiology, as well as pathological conditions, including neuropsychiatric diseases, such as Alzheimer’s disease, schizophrenia, Parkinson’s disease, and MDD [22,23,24,25,26]. In MDD, miRs act at different levels in the process of neurogenesis and synaptic plasticity, by regulating specific genes that are critical components of various signaling pathways involved in MDD development [27,28]. Being ubiquitously found in numerous body fluids, such as blood, saliva, urine, tears, and cerebrospinal fluid, or even encapsulated in small vesicles (exosomes) [29], they have the great advantage of being easy to collect and analyze. In addition, miRs appear to have a chemically superior stability compared to other RNA-based molecules, such as mRNA, and their expression has been found to be dysregulated in MDD patients when compared to healthy controls. Therefore, taken together, the minimally invasive screening technique of circulating miRs in biological samples of MDD patients might represent a promising tool for the accurate and optimized diagnostic and treatment response monitoring of this neuropsychiatric illness [26,28,29,30,31].

Therefore, the aim of this review article is to critically and systematically review the differences in miR levels in MDD patients vs. controls, at baseline and before and after antidepressant treatment and their potential diagnostic and therapeutic relevance in the context of biomarker development, for a better characterization and reliable diagnosis of MDD, which could open novel horizons in the field of neuropsychiatry.

## 2. Materials and Methods

### 2.1. Search Strategy and Study Selection

All research articles included in this study were retrieved by two independent investigators by interrogating the PubMed, Web of Knowledge, and DirectScience databases (up to 20 of March 2021) with the following combination of key words: (“depression” or “depressive disorder”), and (“microRNA” or “miR”), and (“blood compartments”), and (“diagnosis”), and (“treatment” or “antidepressant treatment” or “antidepressant” or “therapy”), and (“biomarker”). The references from the articles of interest were analyzed to identify other relevant reports.

After the electronic search, duplicate references were excluded. Titles, abstracts, and study methodologies were screened based on the inclusion and exclusion criteria.

### 2.2. Inclusion and Exclusion Criteria

We selected studies that contained patients with diagnosed MDD and healthy controls. The index tests for candidate miRs from the selected specimen types (blood compartments) were performed by quantitative real-time PCR. Patients were diagnosed with MDD based on the Structured Clinical Interview for DSM-IV Axis I Disorders (SCID-I) (reference standard).

Research articles’ inclusion criteria were: (1) case-control studies in human subjects on depression assessing miRs’ expression level, with or without AD, (2) studies evaluating the diagnostic potential of different miRs in MDD, (3) MDD diagnosed based on the Structured Clinical Interview for DSM-IV Axis I Disorders (SCID-I), (4) a control group consisting of healthy subjects, and (5) published in the English language.

Research articles’ exclusion criteria were: (1) studies not conducted on human subjects, (2) studies assessing miR expression in body fluids other than blood, (3) nonoriginal papers, such as conference abstracts, letters to editors, and reviews, (4) duplicate studies, and (5) papers not written in the English language.

Disagreement between the two reviewers (D.N. and C.H.) was resolved by discussion. Our study protocol was not prospectively registered.

### 2.3. Data Collection and Characteristics of the Included Studies

We further considered for analysis only research articles that presented data related to the screening and validation of miRs in MDD from blood compartments (whole blood, serum, total plasma (TP), plasma exosomes, exosome-depleted plasma (EDP), and peripheral blood mononuclear cells (PBMCs)). Extracellular vesicle (EV)-entrapped miRs, such as in exosomes, have also been explored as sources of biomarkers for MDD.

**Figure 1:**
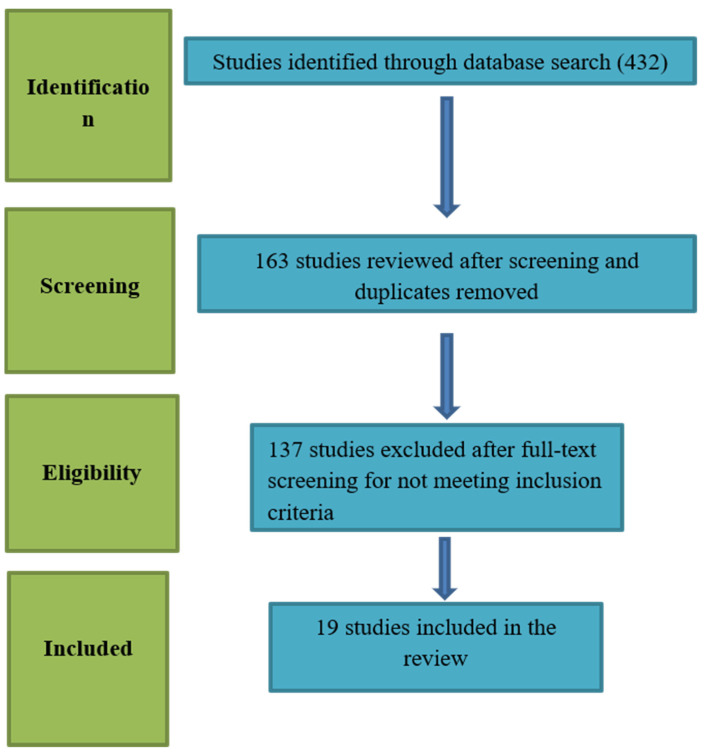
Flow diagram of the study selection process.

From the initial search, 432 articles were found. After title and abstract screening, a total of 163 articles were included. After full-text reading, 137 studies were excluded, and 19 articles were included for the present paper. Figure 1 summarizes our study selection process.

## 3. Results

There is a high heterogeneity regarding the analyzed miRs in MDD, in peripheral blood compartments. The included studies revealed dysregulations in the expression levels of miRs in depressed patients compared to healthy controls, as well as before and after AD. Methodological and study design differences were observed among the studies, which can explain the heterogeneity of the analyzed miRs. Not all authors specified the analyzed blood compartment; the specified blood compartments included: whole blood, plasma, serum, and PBMCs.

Table 1 presents for each study the sample size (number of cases/controls), the blood compartment used for the analyses (some authors did not specify this), and miR findings and expression (upregulated, downregulated, or unchanged miRs) in depressive patients compared to healthy controls.

A total number of 106 miRs were identified in the aforementioned studies, out of which 49 were upregulated and 35 downregulated and 22 presented no changes in expression levels (MDD patients vs. healthy controls). The most frequently upregulated miRs were miR-107, miR-132, miR-182, and miR-124, while the most frequently downregulated were miR-381 and miR-451a. There were inconsistencies regarding miR-107, as some studies found it upregulated, others downregulated, or even unchanged in MDD patient samples vs. controls.

Table 2 shows the 49 upregulated miRs and the 35 downregulated miRs in patients with MDD (compared to controls).

Table 3 shows the sample characteristics for each study investigating miRs before and after AD in MDD patients, the blood compartment used for the analyses, and miR findings and expression level changes in depressive patients, before and after AD.

A total number of 88 different miRs were identified in relation to treatment response. There were 61 miRs upregulated and 26 downregulated in MDD patients as a response to AD. We found inconsistent reports regarding several miRs and one unchanged miR (miR-26b) after AD. The most prevalent upregulated miRs after AD treatment were let-7e, miR-183, and miR-335.

There is an increasing interest regarding miR changes towards treatment response. Several antidepressants exert their effects by targeting miRs. This suggests that miRs may be used as biomarkers for monitoring therapy response in patients with MDD. The treatment regimens included in the present studies were mostly SSRI (citalopram, paroxetine, sertralinum) and SNRI (venlafaxine, duloxetine). The average period of treatment was eight weeks. Some analyses were performed after four weeks of treatment, while in other studies, after ten weeks or twelve weeks.

Interestingly, the majority of miRs studies changed their expression pattern after AD treatment, but some maintained their expression level. This is the case of miR-494, -589, -26b, -34a-5p, -124, and -132, which remained upregulated even after treatment, while miR-451a remained downregulated after treatment.

Table 4 presents the common miRs found in MDD patient samples and their expression level before and after antidepressant administration.

## 4. Discussion

miRs can be detected in different body fluids and in various blood compartments, such as whole blood, plasma, serum, and PBMCs. They have been discovered to have a regulatory role, both in physiological activities and in the pathogenesis of certain diseases. Recent literature reports suggest that miRs may influence the etiology and pathophysiology of psychiatric disorders, including MDD [32].

Mounting evidence suggests that a tremendous number of miR species possess a dysregulated expression pattern in MDD patients relative to healthy controls. miR-132 was among the top-ranked upregulated miRs within the studies, with evidence demonstrating its direct involvement in the pathophysiology of MDD. Animal studies have shown that the increase in miR-132 expression negatively correlated with brain-derived neurotrophic factor (BDNF) expression and that inhibiting miR-132 leads to an increase in BDNF expression and to the reduction of depression symptoms. Moreover, a high miR-132 expression level leads to short-term memory and learning impairment [33].

On the other hand, the most frequently downregulated miRs in MDD patients vs. controls were miR-381 and miR-451a, which are not very well documented to date. It is known, however, that miR-451a downregulates the expression of the macrophage migration inhibitory factor in cells, but its exact mechanistic role in MDD development is still under investigation [38].

Another upregulated miR, miR-155, was also found among the included studies, and mechanistically, it was shown that it represses the expression of the silent information regulator 1 (SIRT1) gene, involved in the circadian rhythm and depression [46].

In addition, some miRs kept their expression levels constant even after administration of AD treatment (let-7e, miR-183, and miR-335); however, contradictory studies exist, and their exact role in MDD etiopathogenesis is yet to be understood [51,52].

Another miR that is well documented in the literature in relation to depression is miR-182. Animal studies performed on rats showed that suppressing miR-182 expression in the hippocampus exerts antidepressant-like effects, thus suggesting that this miR is directly involved in the development of MDD. Overexpression of miR-182 increased the symptoms of depression and decreased the BDNF levels [53]. miR-124 is also involved in the development of MDD and was found by us as one of the most frequently upregulated miRs in patient samples. Wang et al. (2017) showed that, in HEK 293 cell lines, miR-124 targeted the glucocorticoid receptor (GR). Moreover, miR-124 suppression leads to activation of BDNF and induced behavioral improvement of mice, thus suggesting a potential biomarker and therapeutic target for AD drug development, as also suggested by another review report [54,55].

Interestingly, other miRs have been shown to have an inconsistent dysregulation pattern among studies. For example, miR-16 and miR-135a were found to be either upregulated or downregulated, depending on the cohorts examined, the phenotype, and the tissue specimen. This outcome could also possibly occur due to variables that were not taken into account in the literature reports. Both miRs regulate the expression of the serotonin transporter (SERT). Therefore, independent validation of significantly dysregulated individual miRs in blood samples of MDD patients could aid in the biomarker development for the diagnostics of MDD, enriching current diagnostic strategies that remain only moderately reliable to date [9].

Furthermore, we observed that numerous miRs changed their expression pattern after AD. One explanation could be that some miRs are directly involved in the mechanism of action of SSRI and SNRI drugs. Of note, miR-16 targets the SERT and acts as a central effector in mediating the adaptive response to treatment with fluoxetine, of serotoninergic and noradrenergic neurons [56].

Taken together, these studies demonstrate that circulating miRs have a great diagnostic biomarker potential for MDD detection. Moreover, the discovery of altered miR levels between circulating samples of MDD patients compared to healthy controls could lead to a better understanding of the molecular mechanisms involved in the complex neurobiology of this debilitating disorder. Validation of independent miR candidates in individual cohort studies is another crucial step for biomarker development in order to increase the confidence of the results. In addition, as many miRs change their expression pattern after administration of AD, they represent promising candidates not only for treatment response monitoring indicators, but also for the discovery of unknown mechanisms of action.

However, our review has some limitations in assessing the use of miRs as biomarkers of MDD, as some studies are inconsistent and/or conflicting regarding miR expression levels. This heterogeneity could primarily arise due to technical and methodological differences regarding sample types and collection, processing, RNA extraction, and the choice for further downstream statistical analysis. In order to increase the specificity of potential diagnostic biomarkers of MDD, the use of different control groups would be required. However, the latter strategy might lead to difficulties in comparisons between different studies for review reports.

## Figures and Tables

**Table 1:** miRs’ expression in different blood compartments of patients with MDD compared to healthy controls.

Study (Year, Reference No)	Patients	Controls	Blood Compartment	Upregulated miRs	Downregulated miRs	Unchanged miRs	Total
Belzeaux et al., 2012 [32]	16	13	PBMCs	miR-107 miR-133a miR-148a miR-425-3p miR-494 miR-579 miR-652 miR-941 miR-589	miR-200c miR-381 miR-571 miR-636 miR-1243	-	9 upregulated, 5 downregulated
Li YJ et al., 2013 [33]	40	40	Serum	miR-132 miR-182	-	-	2 upregulated
Fan et al., 2014 [34]	81	46	PBMCs	miR-26b miR-1972 miR-4485 miR-4498 miR-4743	-	-	5 upregulated
Li J et al., 2015 [35]	18	18	Whole blood	miR-644 miR-450b miR-328 miR-182	miR-335 miR-583 miR-708a miR-650 miR-654a	miR-541 miR-663 miR-578	4 upregulated, 5 downregulated, 3 unchanged
Camkurt et al., 2015 [36]	50	41	Plasma	miR-451a miR-17-5p miR-223-3p	miR-320a	miR-25-3p miR-126-3p miR-16-5p miR-93-5p	3 upregulated, 1 downregulated, 4 unchanged
Wan et al., 2015 [37]	38	27	Serum	let-7d-3p miR-34a-5p miR-221-3p miR-125a-5p miR-30a-5p miR-29b-3p miR-10a-5p miR-375 miR-155–5p miR-33a-5p miR-139–5p	miR-451a miR-15b-5p miR-106-5p miR-590-5p miR-185-5p	-	11 upregulated, 5 downregulated
Wang X et al., 2015 [38]	169	52	Plasma	-	miR-144-5p	-	1 downregulated
Mafioletti et al., 2016 [39]	20	20	Peripheral venous blood	hsa-miR-199a-5p hsa-miR-24-3p hsa-miR-425-3p hsa-miR-29c-5p hsa-miR-330-3p hsamiR-345-5p	hsa-let-7a-5p hsa-let-7d-5p has-let-7f-5p has-miR-1915-3p	hsa-miR-720 hsa-miR-140-3p hsa-miR-1973 hsa-miR-30d-5p hsa-miR-3158-3p hsa-miR-330-5p hsa-miR-378a-5p hsa-miR-1915-5p hsa-miR-1972 hsa-miR-21-3p hsa-miR-4521 hsa-miR-4793-3p hsa-miR-4440	6 upregulated, 4 downregulated, 13 unchanged
Sun et al., 2016 [40]	32	32	Peripheral blood leukocytes	miR-34b-5p miR-34c-5p	-	miR-369–3p miR-381 miR-107	2 upregulated, 3 unchanged
He et al., 2016 [41]	32	30	PBMCs	miR-124	-	-	1 upregulated
Roy et al., 2017 [42]	18	17	Serum	miR-124-3p	-	-	1 upregulated
Kuang et al., 2018 [43]	84	78	Serum	miR-34a-5p miR-221-3p	miR-451a	-	2 upregulated, 1 downregulated
Fang Y et al., 2018 [44]	45	32	Plasma	miR-124 miR-132	-	-	2 upregulated
Gheysarzadeh et al., 2018 [45]	39	36	Serum	-	miR-16 miR-135a miR-1202	-	3 downregulated
Hung et al., 2019 [46]	84	43	PBMCs	miR-21-5p miR-145 miR-223	miR-146a miR-155 let-7e	-	3 upregulated, 3 downregulated

**Table 2:** Upregulated and downregulated miRs in MDD patients’ vs. healthy controls from all studies included in this review (miR names in bold were identified by more than one independent study).

Upregulated miRs	Downregulated miRs
**miR-107**	miR-200c
miR-133a	**miR-381**
miR-148a	miR-571
miR-425-3p	miR-636
miR-494	miR-1243
miR-579	hsa-let-7f-5p
miR-652	hsa-miR-1285-5p
miR-941	hsa-miR-107
miR-589	hsa-miR-26a-5p
hsa-miR-5010-3p	hsa-miR-26b-5p
hsa-miR-151a-3p	brain-miR-161
**miR-132**	brain-miR-112
**miR-182**	hsa-let-7d-3p
miR-26b	hsa-miR-103a-3p
miR-1972	hsa-miR-532-5p
miR-4485	miR-335
miR-4498	miR-583
miR-4743	miR-708a
miR-644	miR-650
miR-450b	miR-320a
miR-328	miR-15b-5p
miR-451a	miR-106-5p
miR-17-5p	miR-590-5p
let-7d-3p	miR-185-5p
miR-223-3p	miR-144-5p
miR-34a-5p	hsa-let-7a-5p
miR-221-3p	hsa-let-7d-5p
miR-125a-5p	has-let-7f-5p
miR-30a-5p	has-miR-1915-3p
miR-29b-3p	**miR-451a**
hsa-miR-199a-5p	miR-16
hsa-miR-24-3p	miR-135a
hsa-miR-425-3p	miR-1202
hsa-miR-29c-5p	miR-146a
hsa-miR-330-3p	miR-155
miR-10a-5p	let-7e
miR-375	
hsamiR-345-5p	
miR-155–5p	
miR-33a-5p	
miR-139–5p	
miR-34b-5p	
miR-34c-5p	
**miR-124**	
miR-124-3p	
miR-34a-5p	
miR-221-3p	
miR-21-5p	
miR-145	
miR-223	

**Table 3:** miR changes in expression levels before and after antidepressant (AD) treatment.

Study	Patients	AD Treatment and Duration	Blood Compartment	Upregulated miRs	Downregulated miR	Unchanged miRs	Total
Enatescu et al., 2016 [30]	5	Escitalopram 12 weeks	Plasma	miR-1193 miR-3173-3p miR-3154 miR-129-5p miR-3661 miR-1287 miR-532-3p miR-2278 miR-3150a-3p miR-3909 miR-937 miR-676 miR-489 miR-637 miR-608 miR-4263 miR-382 miR-3691-5p miR-375 miR-433 miR-1298 miR-1909 miR-1471	miR-99b miR-151-5p miR-223 miR-181b miR-26a miR-744 miR-301b miR-27a miR-24 miR-146a- miR-146b-5p miR-126 miR-151-3p let-7d miR-221 miR-125a-5p miR-652	-	23 upregulated, 17 downregulated
Li J et al., 2015 [35]	18	Citalopram, 1 week	Whole blood	miR-335	-	-	1 upregulated
Wang X et al., 2015 [38]	169	Not mentioned, 8 weeks	Plasma	miR-144-5p miR-30a-5p	-	-	2 upregulated
He et al., 2016 [41]	32	Venlafaxine (*N* = 7), paroxetine (*N* = 7), fluoxetine (*N* = 3), escitalopram (*N* = 11), duloxetine (N = 1), sertraline (*N* = 3), mirtazapine (*N* = 2)	PBMCs	-	miR-124	-	1 downregulated
Kuang et al., 2018 [43]	84	Paroxetine 8 weeks	Serum	miR-34a-5p miR-221a-3p	miR-451a	-	2 upregulated, 1 downregulated
Fang Y et al., 2018 [44]	32	Citalopram 8 weeks	Plasma	miR-124	miR-132	-	1 upregulated, 1 downregulated
Hung YY et al., 2019 [46]	84	Not mentioned, 4 weeks	PBMCs	miR-146a miR-155 let-7e	-	-	3 upregulated
Bocchio-Chiavetto et al., 2013 [47]	10	Escitalopram 10 weeks	Whole blood	miR-130b miR-505 miR-29b-2 miR-26b miR-22 miR-26a miR-64 miR-494 let-7d let-7g let-7e let-7f miR-629 miR-106b miR-103 miR-191 miR-128 miR-502-3p miR-374b miR-132 miR-30d miR-500 miR-589 miR-183 miR-574-3p miR-140-3p miR-335 miR-361-5p	miR-34c-5p miR-770-5p	-	26 upregulated, 2 downregulated
Zhang et al., 2014 [48]	20	Venlafaxine, sertraline, mirtazapine 6 weeks	PBMCs	-	miR-1972 miR-4485 miR-4498 miR-4743	miR-26b	4 upregulated, 1 downregulated
Lopez et al., 2017 [49]	23	Escitalopram 8 weeks	Peripheral blood	miR-1202	-	-	1 upregulated
Lin CC et al., 2018 [50]	33	Not mentioned, 4 weeks	Whole blood	miR-16 miR-183 miR-212	-	-	3 upregulated

**Table 4:** Expression level of common miRs before and after AD treatment.

Common miRs in MDD (No Treatment and After ADT)	miRs of MDD Patients Before Antidepressant Treatment vs. Controls	miRs of MDD Patients After Antidepressant Treatment vs. Controls
let-7d	up, down	up, down
let-7e	down	up
let-7f	down	up
miR-16	down	up
miR-24	up	down
miR-26a	down	up, down
miR-26b	up	up
miR-29b	up	up
miR-30a-5p	up	up
miR-34a-5p	up	up
miR-34c-5p	up	down
miR-106b	down	up
miR-124	up	up, down
miR-125a-5p	up	down
miR-132	up	up, down
miR-144-5p	down	up
miR-146a-3p	down	up, down
miR-151-3p	up	down
miR-155	up, down	up, down
miR-221	up	up, down
miR-223	up	down
miR-335	down	up
miR-375	up	up
miR-425-3p	up	down
miR-451a	up, down	down
miR-494	up	up
miR-532	down	up
miR-589	up	up
miR-652	up	down
miR-1202	down	up
miR-1972	down	down
miR-4485	up	down
miR-4498	up	down
miR-4743	up	down

## Data Availability

All data is available in the manuscript.
